# Pheochromocytoma crisis with refractory Acute Respiratory Distress Syndrome (ARDS), Takotsubo syndrome, emergency adrenalectomy, and need for Extracorporeal Membrane Oxygenation (ECMO) in a previously undiagnosed and asymptomatic patient, due to the use of metoclopramide

**DOI:** 10.1186/s12902-023-01404-4

**Published:** 2023-07-10

**Authors:** Yuhua Xie, An Zhang, Min Qi, Bin Xiong, Suhua Zhang, Jianzhong Zhou, Yunxing Cao

**Affiliations:** 1 Department of Critical Care Medicine, The Second Affiliated Hospital of Chongqing Medical University, Chongqing, China; 2 Department of Endocrinology, The First Affiliated Hospital of Chongqing Medical University, Chongqing, China; 3 Department of Cardiovascular Medicine, The First Affiliated Hospital of Chongqing Medical University, Chongqing, China

**Keywords:** Acute respiratory distress syndrome, Critical care ultrasonic examination, Emergency adrenalectomy, Pheochromocytoma crisis, Veno-arterial extracorporeal membrane oxygenation

## Abstract

**Background:**

Pheochromocytoma (PCC) crisis is a rare life-threatening endocrine emergency. The diagnosis and treatment of PCC crisis, with acute respiratory distress syndrome (ARDS) as the first manifestation, is highly challenging, and traditional PCC management strategies are no longer suitable for these patients.

**Case presentation:**

A 46-year-old female patient was admitted to the Intensive Care Unit (ICU) following sudden-onset acute respiratory distress and subsequent initiation of mechanical ventilation via endotracheal intubation. She was initially suspected of having a PCC crisis through the bedside critical care ultrasonic examination protocol. The computed tomography examination revealed a left adrenal neoplasm of 6.5cm × 5.9cm. The plasma-free metanephrine level was 100 times higher than the reference value. These findings were compatible with her PCC diagnosis. Alpha-blockers and fluid intake were started immediately. The endotracheal intubation was removed on the 11th day after admission to the ICU. The patient progressed to severe ARDS again, and invasive ventilation and continuous renal replacement therapy were needed. Despite aggressive therapy, her condition deteriorated. Therefore, she underwent veno-arterial extracorporeal membrane oxygenation (VA-ECMO)-assisted emergency adrenalectomy after multidisciplinary discussion. Postoperatively, the patient was supported by VA-ECMO for 7days. She was discharged from the hospital on day 30 after tumor resection.

**Conclusions:**

This case highlighted the challenges in diagnosing and managing ARDS associated with PCC crisis. The traditional preoperative preparation protocol and optimal operation timing for patients with PCC are not suitable for patients with PCC crisis. Patients with life-threatening PCC crisis may benefit from early tumor removal, and VA-ECMO could maintain hemodynamic stability during and after surgery.

## Background

Pheochromocytoma (PCC) is a rare catecholamine-producing tumor arising from chromaffin cells of the adrenal medulla. A recent study showed that the annual incidence of pheochromocytoma was 2–8 cases per million inhabitants [1, 2]. However, no exact data are available on the morbidity or prevalence in China. The predominant symptom is hypertension with target tissue damage, such as hypertrophic or dilated cardiomyopathy [1]. The PCC crisis is a rare, life-threatening endocrine emergency causing the sudden and massive release of catecholamines, which can lead to hemodynamic instability and multi-organ dysfunction [3]. PCC crisis commonly manifests with hypertensive crisis or catecholamine cardiomyopathy [3, 4], while acute pulmonary edema or acute respiratory distress syndrome (ARDS) as the primary manifestation is rare. The mechanism underlying noncardiogenic pulmonary edema and ARDS caused by the PCC crisis is not clear. It may be related to catecholamines contracting venules and lymphatic vessels behind pulmonary capillaries, thus increasing hydrostatic pressure and capillary permeability [5, 6].

Excessive amounts of catecholamines can cause cardiomyopathy. The International Expert Consensus Document on Takotsubo Syndrome proposed that endogenous catecholamine spillover related to pheochromocytoma served as a distinct trigger for Takotsubo syndrome (TTS) [7]. The common symptoms of TTS are acute chest pain, dyspnea, or syncope, which are indistinguishable from those of acute myocardial infarction at first glance. In some patients, TTS may be diagnosed incidentally based on new electrocardiogram changes or a sudden elevation of cardiac biomarkers.

Surgical resection should be performed after the qualitative and localized diagnosis of PCC, but sufficient preparation is necessary before the surgery to prevent perioperative cardiovascular complications [8]. The guideline recommends that patients with symptomatic pheochromocytoma undergo alpha-blockade for 2–4weeks before surgery to normalize their blood pressure and heart rate. The treatments should also include fluid intake and a high-sodium diet to reverse catecholamine-induced blood volume contraction [8, 9]. Regarding the PCC crisis, the timing of the surgery is controversial. Some experts recommend to delay the surgery in patients with PCC crisis when they do not reach a steady state whereas others recommend an emergency tumor removal, which might increase the survival rate [3, 5].

As a new and effective artificial organ replacement support system, extracorporeal membrane oxygenation (ECMO), has saved many lives in treating acute and critical respiratory or circulatory failure and has received increasing attention from clinicians and critically ill patients. However, its clinical application in cardiopulmonary insufficiency caused by PCC is still unclear. ECMO was successfully used to treat refractory cardiogenic shock caused by PCC crisis, and elective adrenalectomy was performed after the cardiopulmonary function was stable [10]. A systematic review reported that 10 patients with phaeochromocytoma-induced cardiogenic shock underwent adrenal resection with extracorporeal life support and achieved good results [11]. However, urgent surgery assisted with ECMO in PCC crisis accompanied by ARDS is rarely reported. Therefore, we reported a patient with a PCC crisis characterized by recurrent ARDS, who underwent emergency adrenalectomy successfully with the assistance of veno-arterial ECMO (VA-ECMO). The patient recovered and was eventually discharged from the hospital.

## Case presentation

**Fig. 1:**
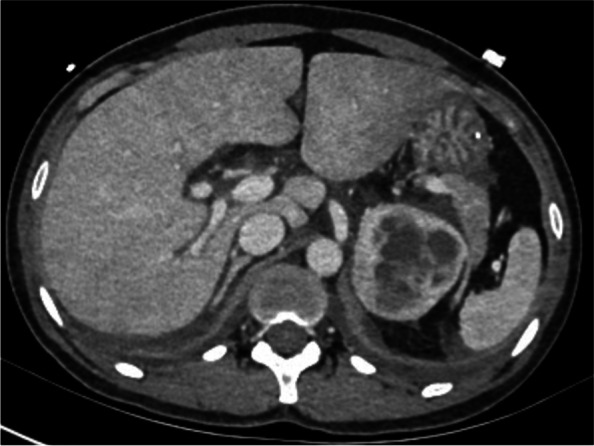
A left-sided adrenal mass of 6.5cm × 5.9cm complicated with tumor stroke was seen on the CT scan

**Fig. 2:**
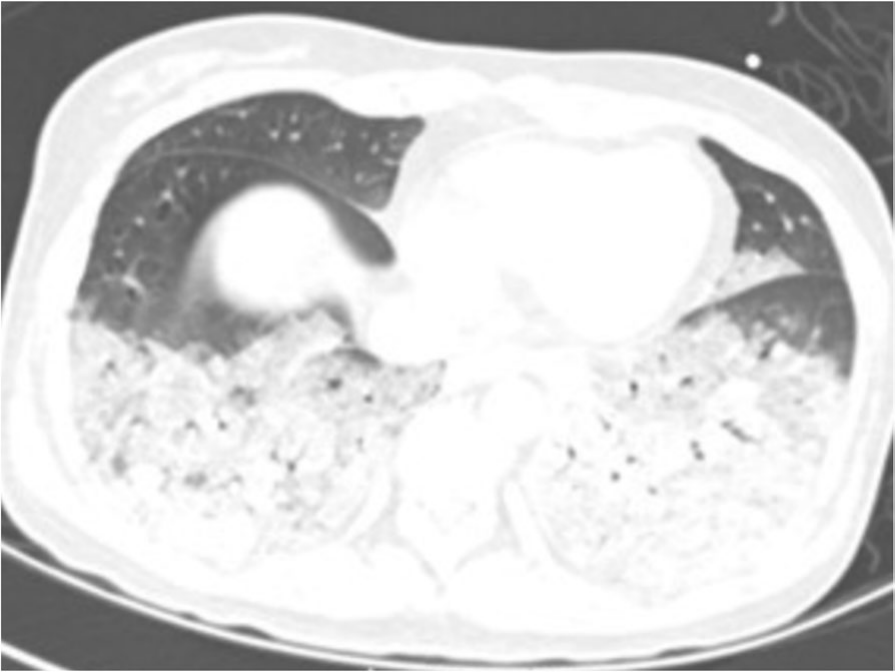
Chest CT showed bilateral diffuse infiltration

**Fig. 3:**
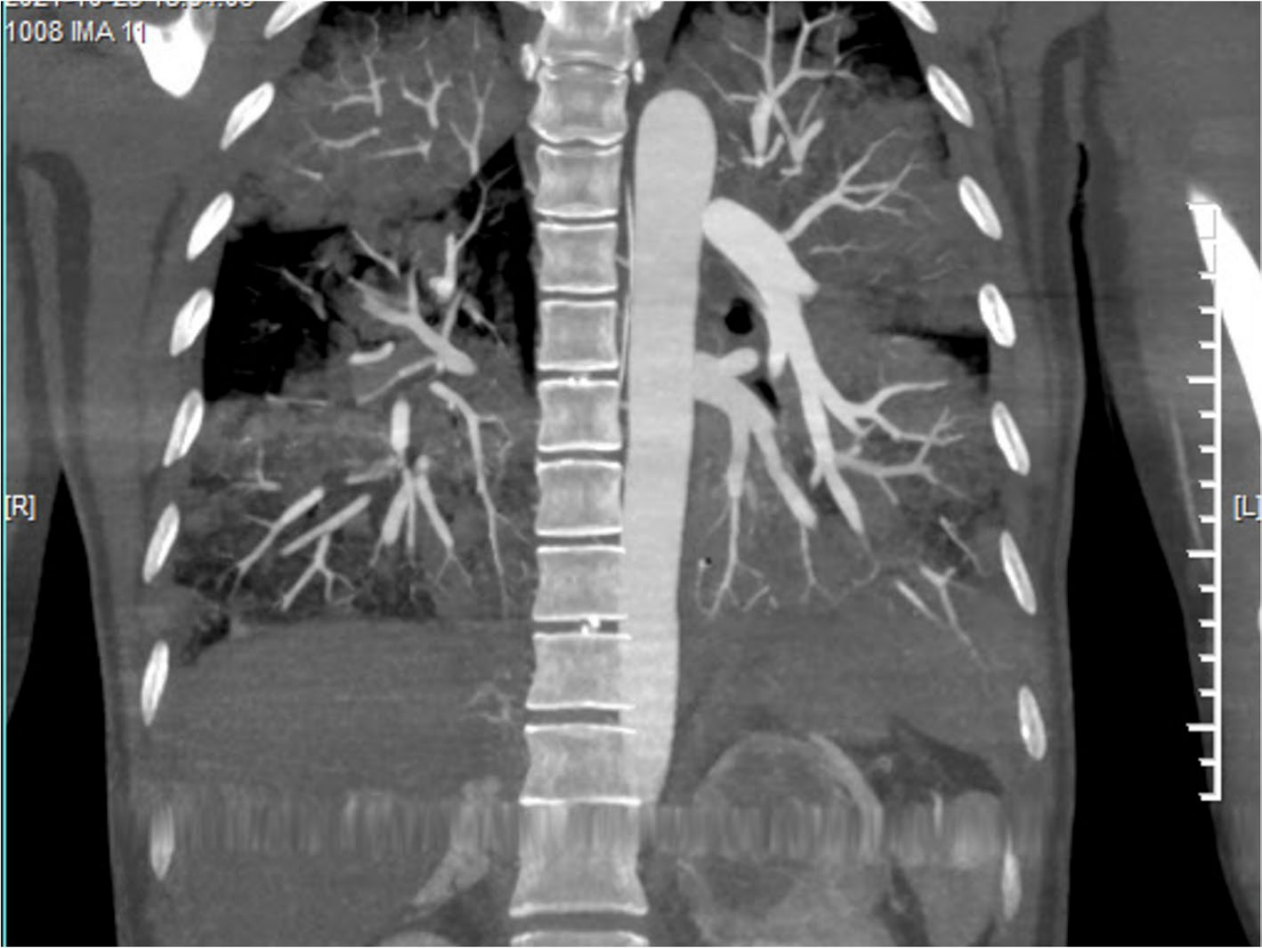
Chest CT showed bilateral diffuse infiltration

A 46-year woman was admitted to our hospital's obstetrics department to remove the intrauterine device (IUD). The patient had previously been in good health and didn't exhibit any fundamental illnesses like hypertension, diabetes, coronary heart disease, headaches, palpitations, sweating, or fainting. On admission, the patient's physical examination revealed a body temperature of 36.5 C, an 80-beat heartbeat, 20 breaths per minute, a 110/82mmHg blood pressure, and no positive findings in the heart, lung, or abdomen physical examination. After admission, routine blood examination, liver function, renal function, and electrocardiogram showed no obvious abnormalities. The patient underwent IUD removal on October 22, 2021, the procedure time was 20min, and the blood pressure was normal before and during the procedure. Nausea and vomiting occurred half an hour and four hours after the procedure, and the symptoms improved after metoclopramide and tropisetron. However, she progressed into respiratory failure, a change of consciousness, and extreme perspiration 8h after the procedure. The clinical examination showed hypoxemia (SaO_2_ 64.7% on room air), tachycardia (140 beats/min), diastolic hypertension (139/126mm Hg), and cyanosis. Diffuse marble spots were seen in the abdomen and thighs, and moist rales were audible over both lungs. The diameter of bilateral pupils was 8mm, with direct and indirect reflections of the light. Arterial blood gas analysis revealed a pH of 7.0, a PaO_2_ of 52mm Hg, a PaCO_2_ of 51mm Hg, a markedly elevated serum lactate level of 17mmol/L, and blood glucose of 38mmol/L. The patient was intubated in the gynecological ward and transferred to the ICU. Fiberoptic bronchoscopy showed that a large amount of watery sputum gushed out from the main airway and lobar bronchi of both lungs. A critical care ultrasonic examination (CCUE) was used to evaluate the cause of respiratory failure immediately after admission to the ICU. The ultrasound examination revealed diffuse B-lines in the entire lung, suggesting severe pulmonary edema. The inferior vena cava collapsibility was 25%, suggesting volume insufficiency, a cardiac output of approximately 2.0 L, and a left ventricular apical wall motion abnormality. No thrombosis was found in the lower limb vein. The abdominal ultrasound showed a mass with an area of about 6.0 × 5.0 cm^2^ in the left splenorenal space. The patient received invasive ventilation with a fraction of inspired oxygen at 100% and continuous renal replacement therapy (CRRT) because of persistent anuria and pulmonary edema. The laboratory data on an emergency basis showed an increased hemoglobin level of 16.3g/dL and leukocytosis of 28.61 × 10^9^/L with a normal C-reactive protein level (< 5mg/L) and 89.0% neutrophils. Her troponin I level was 8.668ng/mL, her myoglobin level 3909.0ng/mL, NT-pro-BNP level was 3549.61pg/mL, her sodium 132mmol/L, and her D-dimer level was greater than 10,000ng/mL. The urine ketone and blood ketone contents were in the normal range. The electrocardiogram showed sinus tachycardia with no change in the ST-T segment. The results of pulse contour cardiac output (PICCO) monitoring showed insufficient circulatory volume, a significantly increased extravascular lung water index of 33mL/kg, significantly increased peripheral vascular resistance, and an increased pulmonary vascular permeability index (9.7; reference interval 1.0–3.0). Based on the aforementioned results, the possible causes of propofol infusion syndrome, air embolism, acute allergic pneumonia, and diabetic ketoacidosis were excluded. The patient presented acute respiratory failure, heart failure, renal failure, consciousness disturbance, acidosis, and abnormal glucose metabolism after gynecological surgery. The levels of cardiac biomarkers increased significantly. Doppler echocardiography showed dyskinesia of the left apex (presenting as left ventricular apical ballooning). The patient had no evidence of infectious myocarditis and acute myocardial infarction. Therefore, we inferred that the patient had Takotsubo syndrome combined with a huge mass in the left splenorenal space. Therefore, we highly suspected that Takotsubo syndrome was induced by pheochromocytoma. Then, we performed abdominal computed tomography (CT) to evaluate the abdominal neoplasm when the patient's vital signs were relatively stable. A left-sided adrenal mass of 6.5cm × 5.9cm complicated with tumor stroke was seen on the CT scan (Fig. 1). Chest CT showed bilateral diffuse infiltration (Figs. 2 and 3). The blood catecholamine hormone test showed extremely elevated levels of metanephrine (3221.27ng/L, reference interval 12–130ng/L), norepinephrine (10,785.34ng/L, reference interval 21–150ng/L), adrenaline (512.63ng/L, reference interval 0–70ng/L), noradrenaline (946.71ng/L, reference interval 65–400ng/L), and dopamine (742.40ng/L, reference interval 0–100ng/L), compatible with massive catecholamine release in PCC. The diagnoses of PCC crisis, Takotsubo syndrome, and multiple-organ dysfunction syndrome were confirmed.

The patient was deeply sedated and treated with fluid resuscitation and a high dose of an alpha-blocking agent (phenoxybenzamine, 20mg t.i.d.) orally. She was weaned from mechanical ventilation on the 11th day after admission in the ICU, and was ready to be transferred to the endocrinology department for preoperative preparation. On the 22nd day after admission to the ICU, the patient had dyspnea again, accompanied by the expectoration of a pink frothy liquid. CCUE screening showed noncardiogenic pulmonary edema; x-rays showed diffuse exudation of both lungs. The patient was diagnosed with ARDS, and invasive ventilation combined with CRRT was performed again. At this time, ICU doctors tended to achieve a negative fluid balance to reduce liquid exudation and protect the patient's pulmonary function. However, the endocrinologists, cardiologists, and urologists suggested continuous fluid intake to stabilize circulation and hence prepare for the surgery because the heart rate of the patient was still fast, about 110bpm. The blood pressure did not drop below 130/80mm Hg, and the skin was dry. The chest x-ray of the patient showed that the pulmonary edema improved after fluid restriction treatment, but the ventilator parameters gradually increased. On the 28th day after admission to the ICU, the patient was in a critical condition. The ventilator parameter settings were as follows: assist/control mode with volume control, the fraction of inspired oxygen 100%, positive end-expiratory pressure 14cm H_2_O, tidal volume 380mL, and peak airway pressure 50cm H_2_O. The P/F ratio continued to be less than 100mm Hg. Our ICU doctor considered whether the patient could be treated by an emergency operation. Therefore, a multidisciplinary discussion was carried out with national experts in endocrinology, urology, cardiology, anesthesiology, and respiratory and critical care medicine. Finally, two treatment schemes were designed: (1) continuing conservative treatment because the patient's vital signs were unstable and surgery had a high mortality rate; (2) emergency surgery because the patient had recurrent ARDS with progressive exacerbation despite medical treatment, suggesting that conventional medical treatment was not effective. If emergency surgery was not performed, the patient might die soon from respiratory failure due to the aggravation of lung injury caused by the continuous release of large amounts of catecholamines from PCC.

**Fig. 4:**
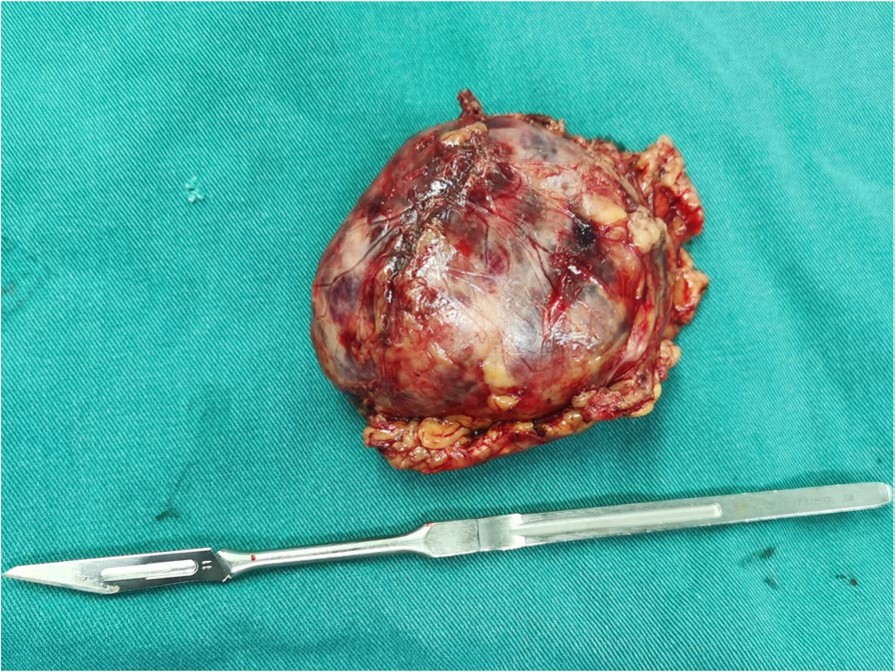
One tumor measuring 6.0cm × 7.0cm was completely resected

**Fig. 5:**
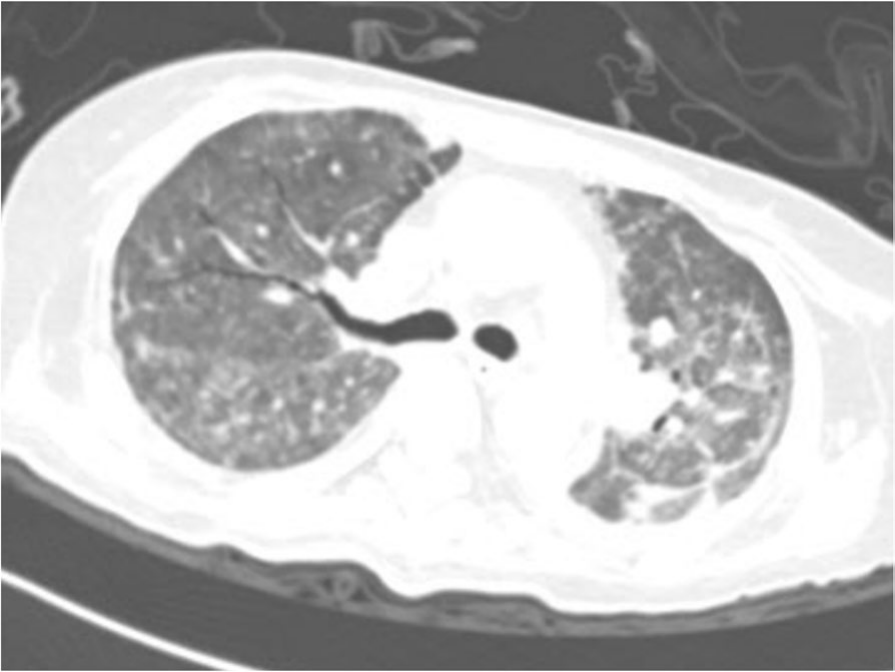
Patient had pulmonary fibrosis when she was discharged from the hospital

**Fig. 6:**
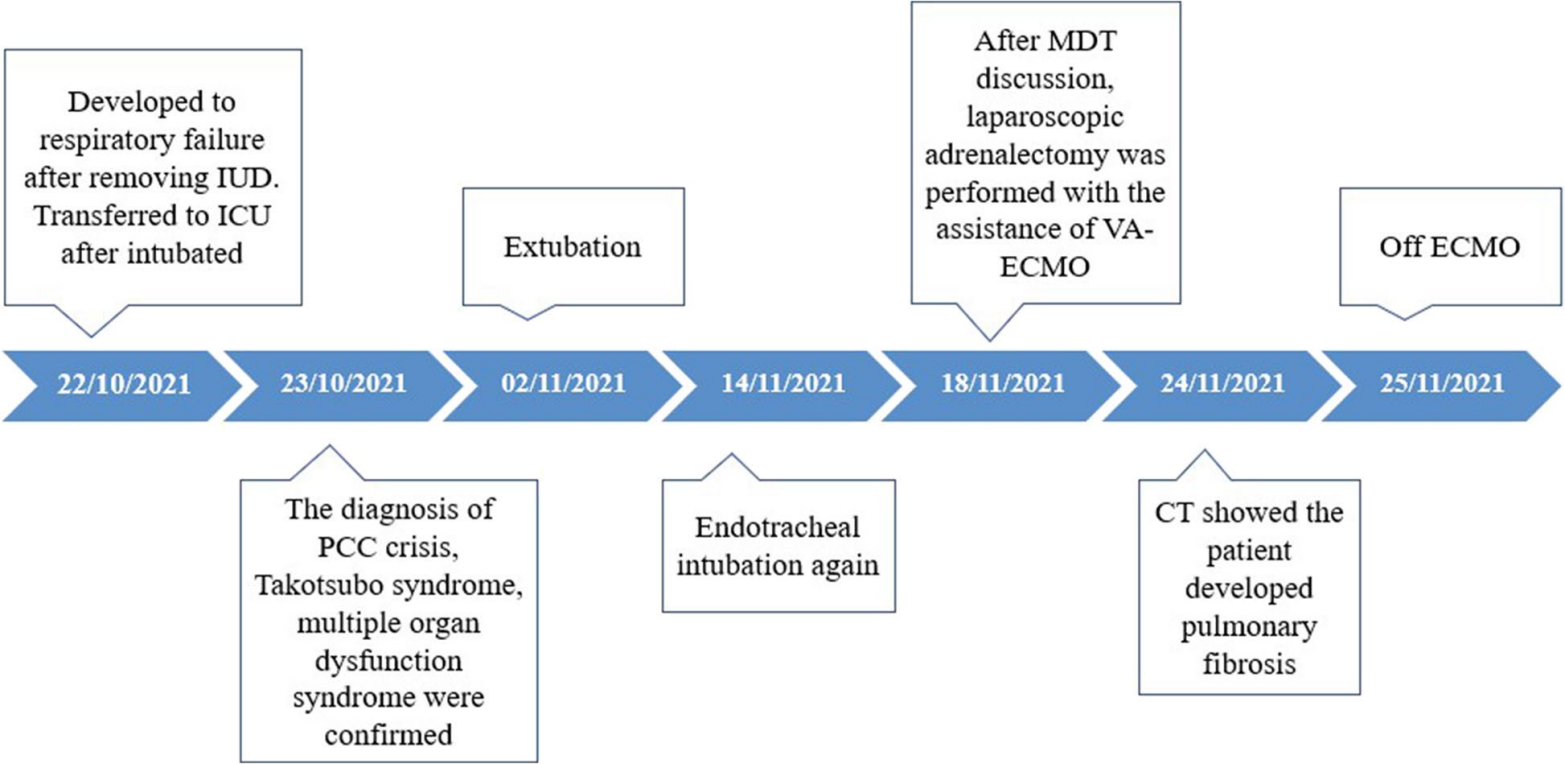
Timeline of case progression

After fully understanding the patient's condition, the patient's family members chose emergency surgical treatment, considering that ECMO could stabilize the patient’s respiratory and circulatory functions during the surgery. The final decision was to perform an emergency adrenalectomy assisted by ECMO. The anesthesiologist pre-configured phentolamine, nitroglycerin, sodium nitroprusside, esmolol, and other drugs to prevent a rapid elevation in intraoperative blood pressure, and pre-configured epinephrine, norepinephrine, dopamine, vasopressin, and other drugs to prevent acute circulatory failure that might occur after tumor resection. An urgent laparoscopic adrenalectomy was performed with the assistance of VA-ECMO, involving skilled anesthesiologists, an abdominal surgeon, and ICU physicians. Her respiratory and circulatory systems were stable during the surgery with the help of VA-ECMO. One tumor measuring 6.0 × 7.0 cm^2^ was completely removed (Fig. 4). Signs of necrosis and hemorrhage were detected inside the tumor, and postoperative pathological biopsy confirmed PCC. The patient was treated with VA-ECMO combined with CRRT and prone-position ventilation after the surgery. Seven days later, VA-ECMO was removed. The mechanical ventilator was removed on day 22 of tumor resection. The levels of catecholamines and metanephrines continued to decrease after operation and returned to normal on the 21st day after the adrenalectomy. The patient recovered and was discharged from the hospital on day 30 postoperatively. Unfortunately, the patient developed pulmonary fibrosis (Fig. 5). Figure 6 shows the patient’s timeline of the clinical condition.

## Discussion

PCC can release large amounts of catecholamines continuously or intermittently, causing persistent or paroxysmal hypertension, multiple-organ dysfunction, and metabolic disorders. The clinical manifestations were mainly headache, palpitation, sweating triad, and serious cases leading to hypertensive crisis, heart failure, cardiomyopathy, and myocardial infarction [1]. However, the patient in our case had an insidious onset. She had no previous history of hypertension, which posed a challenge for diagnosis. The symptoms of elevated diastolic blood pressure, acute pulmonary edema, hyperhidrosis, nausea, vomiting, disturbance of consciousness, pupil changes, leukocytosis, disorder of glucose metabolism, and severe metabolic acidosis in the patient could be explained by PCC. However, the diagnosis of PCC is extremely difficult for patients with no history of hypertension and the onset of acute multiple-organ dysfunction. The critical care physicians quickly screen the causes of dyspnea through the CCUE program and achieve the diagnosis of acute pulmonary edema and Takotsubo syndrome so as to provide clues for the diagnosis of PCC crisis. Thanks to the advancements in critical care ultrasonography technology in recent years, this patient was accurately diagnosed within 24h of admission to the ICU. There have been only a few reported cases of rapid PCC screening through CCUE.

PCC crisis can be triggered spontaneously or by other causes, including tumor touch, trauma, surgical pressure in non-adrenal areas, and certain drugs (such as glucocorticoids, β-blockers, metoclopramide and anesthetics, etc.) [12]. Studies have shown that metoclopramide induces catecholamine release through dopamine receptor antagonists and 5-Hydroxytryptamine receptor 4 [13]. Fentanyl appears to have little effect on histamine release compared to other opioid analgesics such as pethidine, diamorphine, and morphine used in surgical anesthesia. These drugs may promote mast cell histamine release, which in turn triggers catecholamine release from PCC [12]. In this patient, only 0.1mg of fentanyl and 150mg of propofol were used during IUD removal. The patient had nausea and vomiting after surgery but no symptoms of dizziness, palpitation, or hypertension at that time. Metoclopramide was used twice after surgery. Therefore, the possibility of a crisis induced by metoclopramide was considered, but surgical pressure in the non-adrenal area and surgical anesthesia could not be ruled out. Severe acute respiratory syndrome coronavirus 2 (SARS-CoV-2) mRNA or the spike protein may exacerbate catecholaminergic activity, the adrenal glands have been shown to be a significant site of SARS-CoV-2 mRNA accumulation and SARS-CoV-2 spike protein production [14]. The literature has reportedly indicated a connection between the SARS-CoV-2 infection and the PCC crisis [15]. However, the patient was vaccinated with the coronavirus disease 2019 (COVID-19) vaccine 2months before admission. The coronavirus nucleic acid test yielded a negative result, and there were no pre-procedural respiratory symptoms such as fever, cough, or headache, so the patient did not consider COVID-19 or vaccine-induced PCC crisis. However, for patients with PCC or adrenal mass, we should be vigilant to induce PCC crisis when inoculating with the COVID-19 vaccine or diagnosing COVID-19.

Pheochromocytoma can present with TTS. TTS was first named Takotsubo syndrome because the heart shape was similar to that of an octopus fish basket at the time of onset. Later, it was also called stress cardiomyopathy, broken heart syndrome, or apical ballooning syndrome [7, 16]. The 2018 European consensus developed new international diagnostic criteria for the diagnosis of TTS. Patients with TTS have transient left ventricular dysfunction. Midventricular, basal, or focal wall motion abnormalities or apical ballooning, and pheochromocytoma may contribute to the arbitrary syndrome [7]. An excess of catecholamines can cause cardiomyopathy. Catecholamines cause direct damage to the myocardium through calcium overload, oxidative stress, induction of apoptosis, promotion of fibrosis, and activation of the renin–angiotensin–aldosterone system. Catecholamines can also cause indirect damage to the heart via increasing oxygen consumption and decreasing the myocardial oxygen supply, which causes coronary artery spasms, endothelial injury, arrhythmias, and cardiac dysfunction [5, 17, 18]. The elevated cardiac injury markers in our patient could potentially lead to a misdiagnosis of myocarditis or acute myocardial infarction, but thanks to the discovery of apical ballooning in the heart and the mass in the splenorenal space by ultrasound, it provided an important clue for the final diagnosis.

PCC, with pulmonary edema as the first symptom, is uncommon and usually leads to rapid death. It can be seen not only during the course of the disease but also after tumor resection. The causes of pulmonary edema due to PCC can be divided into cardiac and noncardiac causes. In most patients, pulmonary edema is cardiogenic and caused by cardiomyopathy, myocardial infarction, and severe arrhythmias. Noncardiogenic pulmonary edema was thought to be the result of a catecholamine-induced transient increase in pulmonary capillary pressure caused by pulmonary venoconstriction and altered pulmonary capillary permeability [19, 20]. Sukoh reported a patient with pheochromocytoma complicated by acute noncardiogenic pulmonary edema. The bronchoalveolar lavage fluid showed the accumulation of pulmonary neutrophils, suggesting that catecholamines released by PCC might promote the accumulation of neutrophils in the lungs and contribute to the pathogenesis of ARDS [21]. When our patient first developed acute pulmonary edema, the PICCO results showed a significantly increased pulmonary vascular permeability index (PVPI 9.7, reference interval 1.0–3.0). When she developed acute pulmonary edema for the second time, a color Doppler ultrasound showed that the systolic and diastolic functions of the heart were not restrained. It confirmed that the pulmonary edema in this patient was mainly noncardiac, suggesting that catecholamine hormones played a special role in the occurrence and development of pulmonary edema and ARDS. However, the specific pathophysiological basis needs further investigation.

The mortality rate of PCC is reported to be 15%–30% [22]. Functional adrenal tumors are usually an indication for surgery, regardless of their size [8]. Major preoperative preparations include controlling blood pressure with selective or nonselective α-blockers and increasing fluid intake. Surgery should be performed after the patient has achieved blood volume recovery, stable blood pressure, and improvement in hypermetabolic syndrome and glucose metabolism abnormalities [1]. Evidence to determine the best target blood pressure and cardiac status is insufficient. In 2007, the Endocrine Society recommended that the goal be to achieve a target blood pressure of less than 130/80mm Hg while sitting, no less than 80/45mm Hg while standing, and a target heart rate of about 60–0bpm when sitting and 70–80 beats per minute while standing [23]. For severe complications, the time of preoperative preparation should be extended. However, still, no guidelines or expert consensus exists to recommend the timing of surgery for patients in PCC crisis; also, a clear indication of emergency surgery for PCC is lacking. Uchida et al. [24] believed that emergency surgery was feasible when PCC was life-threatening because it could cut off the source of adrenaline release and stabilize the condition quickly. Kakoki reported a 70-year-old patient with PCC crisis who was treated with α-blockers. However, his respiratory status, liver function, and renal function deteriorated progressively. An emergency left adrenalectomy was performed despite his critical condition. The patient was removed from the ventilator support two days after the surgery [25]. If the patient's condition worsened after receiving appropriate treatment for PCC, the tumor needed to be removed urgently, even if the patient was in a critical condition, as this might increase the patient's chances of survival [5]. Thalia Bekelaar [26] showed that seven patients with life-threatening catecholamine crisis underwent emergency adrenalectomy and all survived, suggesting that patients with PCC crisis might benefit from emergency surgery. However, some scholars [3, 27] found that the incidence of perioperative complications and mortality due to emergency surgery were higher than those due to elective surgery, and that emergency surgery should be considered when tumor rupture or uncontrollable bleeding occurs in PCC crisis.

When the patient in our case developed ARDS for the second time, the ventilator support parameters were already high, and the P/F ratio continued to be less than 100mm Hg under pure oxygen inhalation. If the preoperative preparation was done according to the conventional treatment plan for patients with pheochromocytoma, the patients might die of ARDS in a short time. Emergency surgery was the only way to stop the disease progression, but the risk of death in emergency surgery was also quite high with such poor cardiopulmonary function. The tumor size in the patient was huge. The postoperative specimens also found that the tumor had serious internal hemorrhage and necrosis, indicating that the tumor was active, and a large number of catecholamines formed a catecholamine pool inside the tumor. The tumor released a large amount of catecholamines in a short time, leading to the repeated and rapid progress of the patient's disease. Therefore, emergency surgery might be the only way to save the patient's life.

VA-ECMO was used in PCC crisis with severe cardiogenic shock, adrenal resection was performed after hemodynamic stability, and VA-ECMO was removed [28, 29]. However, delayed surgery also increases the risk of recurrent crisis and multi-organ dysfunction, which can be fatal once the disease occurs at this stage. For example, Manita Chudhary [30] reported a patient with PCC crisis who received veno-venous extracorporeal membrane oxygenation (VV-ECMO) treatment for severe ARDS, but soon he developed cardiogenic shock and received VA-ECMO support. VA-ECMO was removed 4days later, but the patient developed severe and progressive ARDS again, requiring 100% oxygen, high positive end-expiratory pressure, and paralysis-prone positioning. He had to be treated with VV-ECMO again. He underwent a VV-ECMO-supported laparoscopic adrenalectomy a week later. The patient's life was saved successfully. However, if the patient underwent VA-ECMO-assisted adrenalectomy immediately after confirming the diagnosis of PCC, he probably wouldn’t have PCC-induced persistent severe ARDS. Emergency surgery assisted by VA-ECMO would greatly reduce the probability of recurrent PCC crisis, medical costs, and length of hospital stay.

In our patient, the tumor was successfully removed with the assistance of VA-ECMO. The intraoperative blood pressure and oxygen saturation were basically stable, with no intractable hypotension after tumor resection, indicating that patients in PCC crisis could benefit from emergency adrenal resection assisted by VA-ECMO. Unfortunately, our patient had pulmonary fibrosis when she was discharged from the hospital. It also suggested that the timing of surgery for our patient could actually be earlier, such as when the PCC crisis was identified or when the ARDS was stable, and the patient could be extubated for the first time. However, this strategy must be carefully considered because patients with complete heparinization have a high risk of bleeding at the surgical site. The clinical value of VA-ECMO-assisted emergency adrenalectomy still needs more prospective studies for confirmation.

## Conclusions

This case highlighted the challenges in diagnosing and managing ARDS associated with the PCC crisis. The traditional preoperative preparation plan and the operation timing for patients with PCC are not suitable for patients with PCC crisis. Delaying surgery to achieve organ stability may lead to recurrent crisis and multiple-organ dysfunction or even death. Patients with life-threatening PCC crisis may benefit from emergency adrenal resection, and VA-ECMO can maintain hemodynamic stability during and after emergency surgery. At present, guidelines and expert consensus on the management of PCC crisis are lacking, and relevant guidelines and expert consensus need to be urgently formulated to guide clinical treatment.

## Data Availability

All data generated or analysed during this study are included in this published article.

## Abbreviations

PCC: Pheochromocytoma
ARDS: Acute respiratory distress syndrome
ICU: Intensive care unit
ECMO: Extracorporeal membrane oxygenation
VA- ECMO: Veno-arterial extracorporeal membrane oxygenation
TTS: Takotsubo syndrome
IUD: Intrauterine device
CCUE: Critical care ultrasonic examination
CRRT: Continuous renal replacement therapy
PICCO: Pulse contour cardiac output
SARS-CoV-2: Severe acute respiratory syndrome coronavirus 2
COVID-19: Coronavirus disease 2019
VV-ECMO: Veno-venous extracorporeal membrane oxygenation
